# Disparity in anterior cruciate ligament injury management: a case series review across six National Health Service trusts

**DOI:** 10.1186/s12891-025-08572-5

**Published:** 2025-04-15

**Authors:** Niall J. Maher, Chris Brogden, Anthony C. Redmond, Heidi J. Siddle, Gareth Jones, Damian Buck, Steven Broadbent, Gareth Liversidge, Justin Murr, Conor Tingle, David E. Lunn

**Affiliations:** 1 Physiotherapy Department, Leeds Teaching Hospitals NHS Trust, Leeds, UK; 2 Leeds Institute of Rheumatic and Musculoskeletal Medicine, University of Leeds, Leeds, UK; 3 Carnegie School of Sport, Leeds Beckett University, Leeds, UK; 4 School of Health, Leeds Beckett University, Leeds, UK; 5 Bradford Teaching Hospitals NHS Foundation Trust, Physiotherapy, Bradford, UK; 6 Harrogate and District NHS Foundation Trust, Physiotherapy, Harrogate, UK; 7 Calderdale and Huddersfield NHS Foundation Trust, Physiotherapy, Huddersfield, UK; 8 Airedale NHS Foundation Trust, Physiotherapy, Keighley, UK; 9 Mid Yorkshire Teaching NHS Trust, Physiotherapy, Wakefield, UK

**Keywords:** Using MeSH on demand tool, MeSH terms, Anterior cruciate ligament injuries, Retrospective studies, Patient discharge, Pathways, Outcome assessment, Case series

## Abstract

**Background:**

Effective management of anterior cruciate ligament (ACL) injuries requires a comprehensive approach, from initial assessment, through treatment, rehabilitation, and discharge, however no gold standard care pathway exists to help guide clinicians. This case series provides an overview of current ACL injury management processes in six National Health Service (NHS) Trusts.

**Methods:**

This study utilised a retrospective case series design within six NHS Trusts in the Yorkshire region of the United Kingdom. Using a standard operating procedure, each Trust selected ten consecutive ACL injured patients (≥ 16 years), managed either surgically or non-surgically. Data relating to the patient injury journey, patient and injury characteristics, key pathway events, rehabilitation management, outcome measures, and discharge, were collected. Data was anonymised and analysed using descriptive statistics.

**Results:**

Reviews covered 55 patients, median age 25.5 years, (41 males, 14 females). Median time to specialist assessment from injury was 12 days (Interquartile Range [IQR] 6 to 20 days), with 43 patients managed operatively, and 12 non operatively. The median number of physiotherapy sessions was 21 (IQR 9 to 29.5), with outcome measures being variably used across Trusts. Trusts using patient reported outcome measures (PROMS) consistently with their patients provided more physiotherapy appointments (34.5 and 27) and achieved higher return to sport (RTS) rates. Time from injury to discharge varied with a median of 421 (IQR 249 to 546) days. Discharge criteria were applied inconsistently across Trusts, with 31% of cases not using specific criteria. However, Trusts using standardised discharge criteria showed better RTS outcomes, with 27 (61%) patients successfully returning to sport.

**Conclusions:**

This case series review highlighted some good practice in initial ACL management across six NHS Trusts in the Yorkshire region. However, from time to MRI diagnosis to discharge, substantial variation in care is observed. Whether treated operatively or non-operatively, for patients aiming to RTS, this was achieved with greater consistency when more physiotherapy appointments were undertaken, outcome measures and PROMs were used, and specific discharge criteria was utilised. Future larger pathway investigation studies incorporating causative and predictive analysis studies on a national scale are required to determine whether similar trends are observed in a wider ACL injured population, which could help to improve national pathways for patients and clinicians working towards ensuring more positive and standardised patient-related ACL injury outcomes.

## Background

Anterior cruciate ligament (ACL) ruptures are a common and problematic injury, resulting in potential long-term knee functional impairment [1], reduced quality of life [2], time lost from sporting activity [3], financial burden [4], and increased risk of secondary osteoarthritis [5]. ACL injuries affect both athletic, and non-athletic populations [6, 7], with increased incidence reported in Australia, Canada, Italy, and New Zealand [8–11] In the United Kingdom (UK) approximately 20,200 ACL injuries occur annually, representing 0.03% of the general population [12]. The rate of ACL surgical reconstructions (ACLR) increased 12-fold between 1997–2017 to 24.2/100000 population, contributing to estimated annual healthcare costs of £63–85 million [13, 14].

Traditionally, a programme of rehabilitation and exercise-based management is undertaken in patients with isolated ACL injuries [15], with ACLR surgery in addition to exercise rehabilitation conducted for those with persistent knee instability, and or concomitant injuries to the knee such as the meniscus, [14, 16]. Physiotherapy rehabilitation is important in successful functional recovery and RTS from ACL injury whether managed operatively or non-operatively[17, 18]. A forty-three patient case series review evaluating a criterion based RTS programme, showed a failure to complete rehabilitation rate of 49%, which has been reported elsewhere in the literature, [19, 20]. Failure to complete rehabilitation may leave the patient more exposed to reinjury, with ACL reinjury rates documented as one in four under the age of twenty-five in athletic patients [21].

Currently no gold standard ACL injury management strategy exists, with substantial variability highlighted in published protocols relating to management, exercise content, duration, and criteria to progress patients [3, 17, 22, 23]. Clinical practice guidelines appear general and demonstrate poor clinical applicability [17], whilst no UK ACL injury management consensus exists [24]. An expert panel of clinicians recently published guidelines on ACL rehabilitation post reconstruction, and whilst agreement was reached, a low level of certainty for most components of rehabilitation was reported [23]. Consequently, significant uncertainty in determining the most effective management strategies for ACL rehabilitation exist, highlighting the need for further evidence-based guidelines.

Criteria including quadriceps and hamstring strength measurements, patient reported outcomes, returning to sport, absence of giving way, and lack of a knee effusion were nominated in a cohort statement required to be met in operative and non-operative ACL injury management [25]. However, there is uncertainty surrounding the use of outcome measures to monitor progress of ACL injured patients [26]. Ninety percent of physiotherapists surveyed consider knee strength to a be a crucial measure before return to sport (RTS), yet only 36% assessed this function; and although 94% believed that rehabilitation 6 − 12 months post-surgery is essential, early patient discharge is common [26]. Failure to meet recognised criteria has been associated with a seven-fold increase in re-injury rate for those who return to high-intensity sports prior to nine months post operatively [27]. As such, the implementation of current ACL injury management strategies in clinical practice is questioned due to inconsistent clinical outcomes and an overall inability to restore functional capacity before discharge [18].

To-date no NHS UK-based studies have investigated current ACL injury operative and non-operative management pathways and management across a number of Trusts in the same local region. Carter and colleagues [28] investigated patients’ perceptions of ACL injury management across multiple sites, finding substantial variability in care, however this was within the same NHS Trust. As such, there is a need to better understand how different NHS Trusts working within the same geographical region manage ACL injured patients. This study aimed to review the ACL injury pathways, management, and discharge processes in six Trusts within the same region. The findings from this case review may contribute to clinical decision making and guide service improvement strategies for ACL injury management.

## Methods

Six UK NHS Trusts contributed cases to this review; Leeds Teaching Hospitals NHS Trust, Airedale NHS Foundation Trust, Bradford Teaching Hospitals NHS Foundation Trust, Calderdale and Huddersfield NHS Foundation Trust, Harrogate and District NHS Foundation Trust, and Mid Yorkshire Teaching NHS Trust. These Trusts were selected to participate as they form the West Yorkshire Association of Acute Trusts (WYAAT), who aim to address health inequalities and deliver joined up acute hospital services to 2.7 million people who live across the West Yorkshire and Harrogate region of the UK. This study was conducted as a retrospective case series review of clinical practice across six NHS Trusts. All patient data were fully anonymised at each site before analysis, with no identifiable data accessed. According to UK Health Research Authority (HRA) guidance, this study did not require NHS Research Ethics Committee (REC) approval or individual patient consent. As no identifiable human data were used, the study was exempt from the informed consent requirements of Article 32 of the Declaration of Helsinki (2013), [29].

Each Trust had their own patient database; however, a standard operating procedure (SOP) was produced and applied to ensure a consistent method of selecting patients for the case review and data extraction in each Trust. At each of the Trusts, a representative of the Adult Physiotherapy team retrospectively extracted the information required from care records for ten adults diagnosed with an ACL injury in their Acute Knee Injury Clinic (AKC), and managed either operatively or non-operatively with rehabilitation. The SOP requested data relating to patient characteristics and injury specifics, key pathway events, rehabilitation management of the ACL injury, and discharge (see Appendix 1 for further detail). To ensure consistency the review only included adults over the age of 16 years who underwent physiotherapy led rehabilitation of ACL injuries (surgical and non-surgical). Patients with multi-ligament injury with or without reconstruction involving both the ACL and posterior cruciate ligament and either or both the lateral collateral ligament and medial collateral ligament, were excluded from this case review due to the reported poor outcomes and different treatment pathways used in these groups, [30].

So as to limit selection bias, a consecutive sampling method was used [31], with ten consecutive patients selected from a specific period defined as the following: care should have commenced three months after each Trust’s services were deemed to have returned to normal after the COVID- 19 closures. Each Trust selected a recognised date that their services resumed and moved forwards on the calendar by three months, then selected the next patients that met the inclusion criteria. To pilot the data extraction procedure, each Trust utilised the SOP to extract data for five patients, to confirm the method of data collection was feasible and produced the required data. Upon confirmation of the SOP’s applicability and suitability data were extracted from a further five patients in all but one Trust. Extracted data included both surgical and non-surgical management of ACL injuries documented during the patient’s care, from injury to discharge for each patient. All data were anonymised within their local Trust before being collated. The data were summarised using descriptive statistics to explore patterns and variation in the care provided across the Trusts.

## Results

Data were extracted from 55 patient records (five Trusts provided data for ten patients, and one provided data for five patients) from six Trusts within the WYAAT collaborative, diagnosed with ACL injuries, from July 2020 to January 2024. The results of the review are displayed in Tables 1 and 2.

**Table 1:** Patient and injury characteristics

Characteristic	Trust 1	Trust 2	Trust 3	Trust 4	Trust 5	Trust 6
Patients *n* = 55^*^	10	10	10	5	10	10
Women *n* (%)	4 (40)	1 (10)	0 (0)	3 (60)	5 (50)	1 (10)
Men *n* (%)	6 (60)	9 (9)	10 (100)	2 (40)	5 (50)	9 (90)
Median Age (Yrs) at time of injury	26	21	25	30	26	24
Sport related *n* (%)	8 (80)	8 (80)	8 (80)	5 (100)	7 (70)	8 (80)
- Contact in sport *n* (%)	0 (0)	0 (0)	0 (0)	1 (20)	2 (28.5)	0 (0)
- Non-contact sport *n* (%)	8 (100)	8 (100)	8 (100)	4 (80)	5 (71)	8 (100)

^*^n denotes the number of patients; (%) denotes the percentage number of patients at each site: Yrs denotes patient age in years

**Table 2:** Summary of pathway, management and discharge

	**Trust 1**	**Trust 2**	**Trust 3**	**Trust 4**	**Trust 5**	**Trust 6**
*Pathway*						
Patients n	10	10	10	5^a^	10	10
Time to first assessment (median days)	12.5	11	12	17	17.5	7
Diagnosis made by physiotherapist (patients)	0	7	10	2	6	4
Diagnosis made by ortho (patients)	10	3	0	3	4	6
Time to MRI scan (mean days)	23.5	17.5	21	40	40	26
Time to patient MRI confirmation (mean days)	35	21.5	57	54	76.5	52.5
Operative management (patients)	6	10	2	5	10	10
Time to surgery (median days)	276.5	212.5	192	87	135	101.5
*Management*						
Protocol Followed (patients)	0	10	6	5	7	10
Limb symmetry index carried out (patients)	5	0	10	5	0	10
Plyometric testing carried out (patients)	5	0	10	5	0	10
Patient Reported Outcomes used (patients)	0	10	0	0	2	10
Number of physiotherapy appointments	11.5	34.5	4	32	16	27
*Discharge*						
Time to discharge (median days)	235.5	633	204	443	526.5	426
Discharge criteria used (patients)	3	0	10	5	0	10
Patient completed rehabilitation (patients)	6	9	3	5	6	10
Patient returned to sport (patients)	3	9	2	3	5	5
Patient returned to service as a result of reinjury (patients)	0	0	0	0	0	0

^a^Trust 4 presented 5 patients

### Patient and injury characteristics

Data collected showed 41 (75%) of the patients reviewed were men, with 14 (25%) women, with a median age of 25.5 years, (interquartile range (IQR) 19.5 to 29.5). Of the 55 patients, 44 sustained sports related injuries, 41 of 55 (75%) were injured during non-contact sporting activities, three of 55 (5%) were sustained in contact situations, and three further patients were injured while at work, with injury specific data not provided in eight cases.

### Key pathway events

**Fig. 1:**
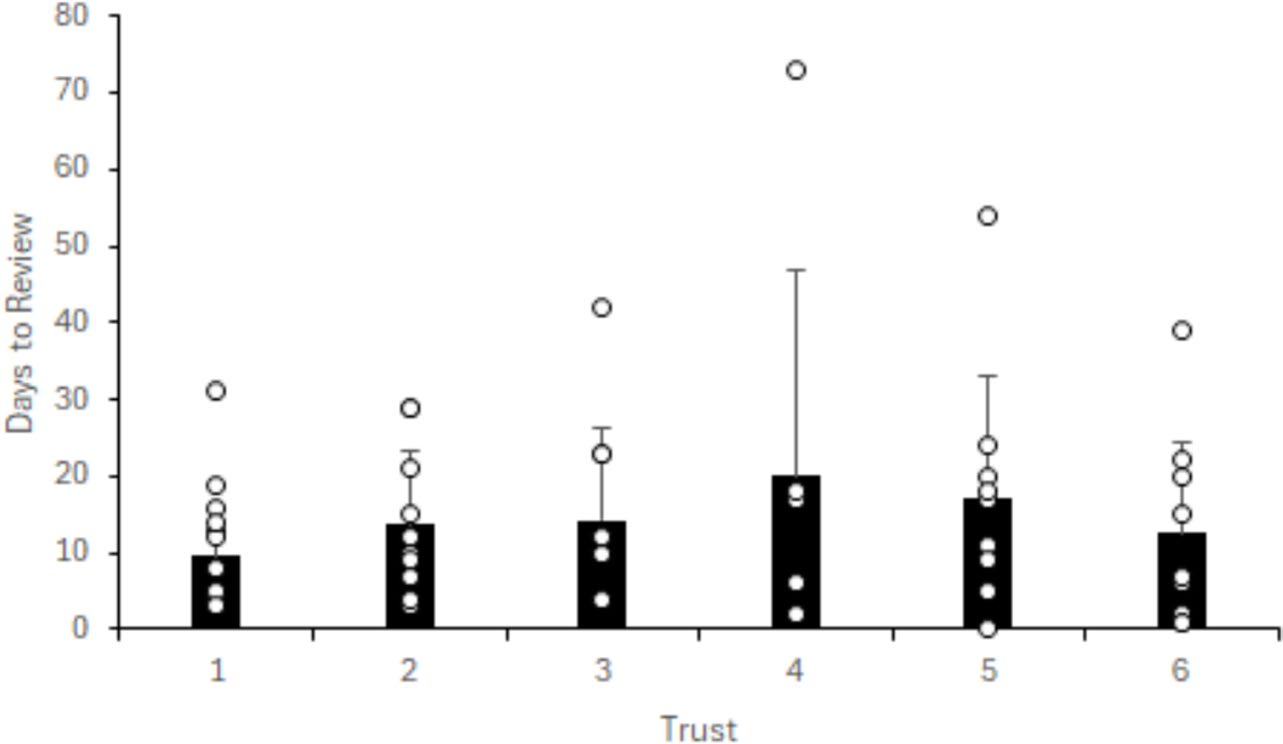
Number of days taken from presenting to hospital to first being seen by an expert clinician/surgeon, according to NHS Trust.* ° denotes each individual patient's numerical data point. For Trusts who presented data for ten patients, less data points are presented if one or more patients presented with the same numerical data point, Trust 4 present five patients*

**Fig. 2:**
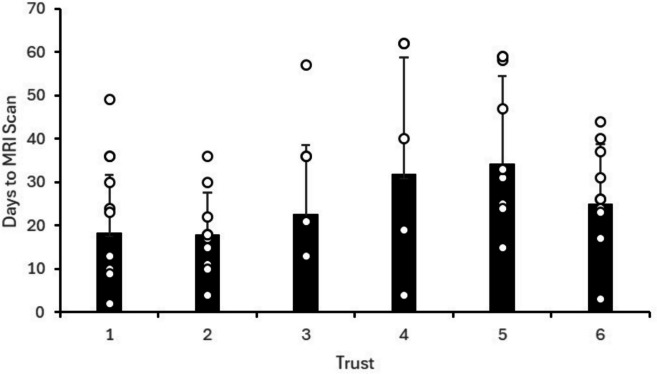
Number of days taken from presenting to hospital to patients first being informed of their MRI results, according to NHS Trust. *° denotes each individual patient's numerical data point. For Trusts who presented data for ten patients, less data points are presented if one or more patients presented with the same numerical data point, Trust 4 present five patients*

Patients were reviewed a median of 12 days (IQR of 6 to 20) after injury, with provisional diagnosis being made by orthopaedic surgeons in 26/55 (47.3%) cases, and physiotherapists in 29/55 cases (52.7%). Figure 1 highlights the variation in patient review data across the six Trusts. All patients across the six Trusts (see Fig. 2) were sent for Magnetic Resonance Imaging (MRI), with findings reported to the patients at a median of 24 days (IQR 15 to 36), and total median wait time of 44 days (IQR 32 to 62) from injury to the patient being made aware of their MRI confirmed diagnosis.

### ACL injury management

**Fig. 3:**
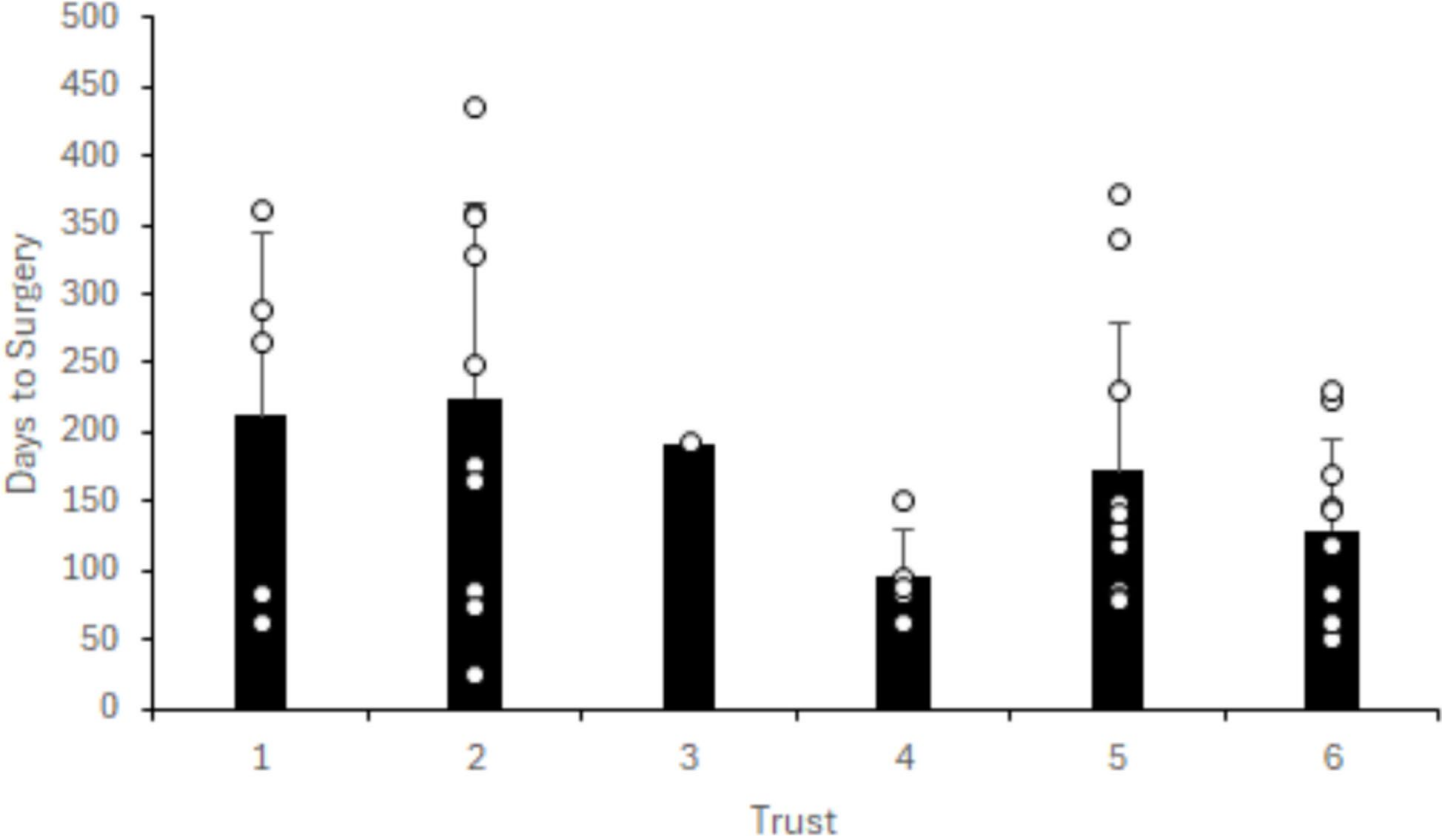
Number of days from first presenting to hospital until surgical intervention according to NHS Trust. *° denotes each individual patient's numerical data point. For Trusts who presented data for ten patients, less data points are presented if one or more patients presented with the same numerical data point or if less patients undertook surgery, Trust 4 present five patients*

**Fig. 4:**
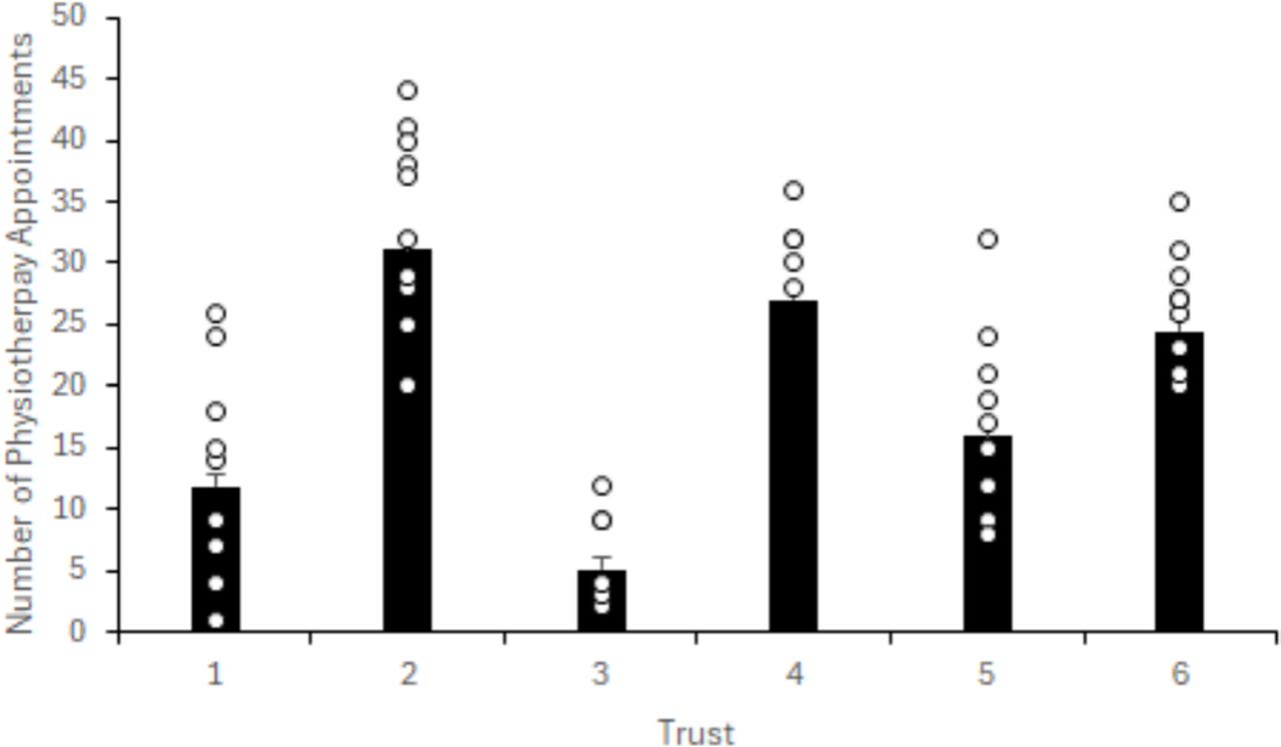
Number of physiotherapy appointments attended by patients according to NHS Trust. *° denotes each individual patient's numerical data point. For Trusts who presented data for ten patients, less data points are presented if one or more patients presented with the same numerical data point, Trust 4 present five patients*

Forty-three (78%) patients underwent ACLR surgery, with three Trusts operating on all ten patients, and one further Trust operating on all five of the patients they presented in their data. Of the twelve patients who were non operatively managed, eight failed to complete their rehabilitation. Median time to surgery from injury was 143 days (IQR 83.5 to 239), with individual patient response data per Trust shown in Fig. 3. Patients attended physiotherapy led rehabilitation classes in 39/55 (71%) cases, with 16 patients (29%) being managed on an individual one to one basis. A specific protocol led rehabilitation regime was used in 42 cases. Patients attended a median of 21 physiotherapy appointments (IQR 9 to 29.5), with Fig. 4 highlighting individual patient appointment data per Trust.

**Fig. 5:**
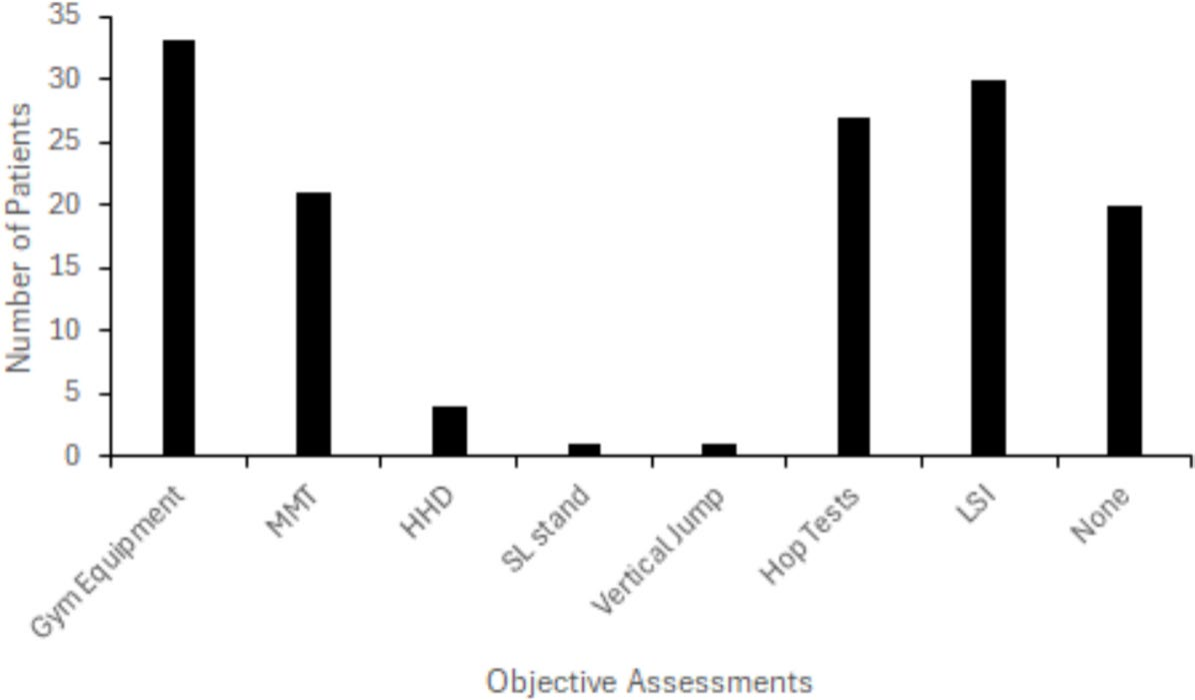
Type and total number of objective measurements used by physiotherapists to inform the rehabilitation process

Plyometric training and testing were used in 30/55 cases (56%). Defined as ratio of results between injured and uninjured limbs expressed as a percentage of symmetry, gym machine-based limb symmetry index (LSI) testing [32] was also used to assess strength progressions in 30/55 cases (56%). Figure 5 highlights the variation in how strength was assessed: with the use of hand-held dynamometer in two cases, manual muscle testing in 11 cases, repetitions to fatigue and capacity testing in six further cases. In 25 cases, it was not documented if or how strength was assessed. No Trust used isokinetic dynamometry to measure strength.

### Discharge

Patient reported outcome measures (PROMs) were used in 22 patients in total, with three Trusts using a cluster of three PROMs with their patients to inform the discharge process. The International Knee Documentation Committee (IKDC) Subjective Knee Form [33] was used in all 22 patients where PROMs were used, followed by the Anterior Cruciate Ligament Return to Sport after Injury Scale (ACL-RSI) [34] in 12 cases, the Tegner activity scale [35] in 10 cases.

**Fig. 6:**
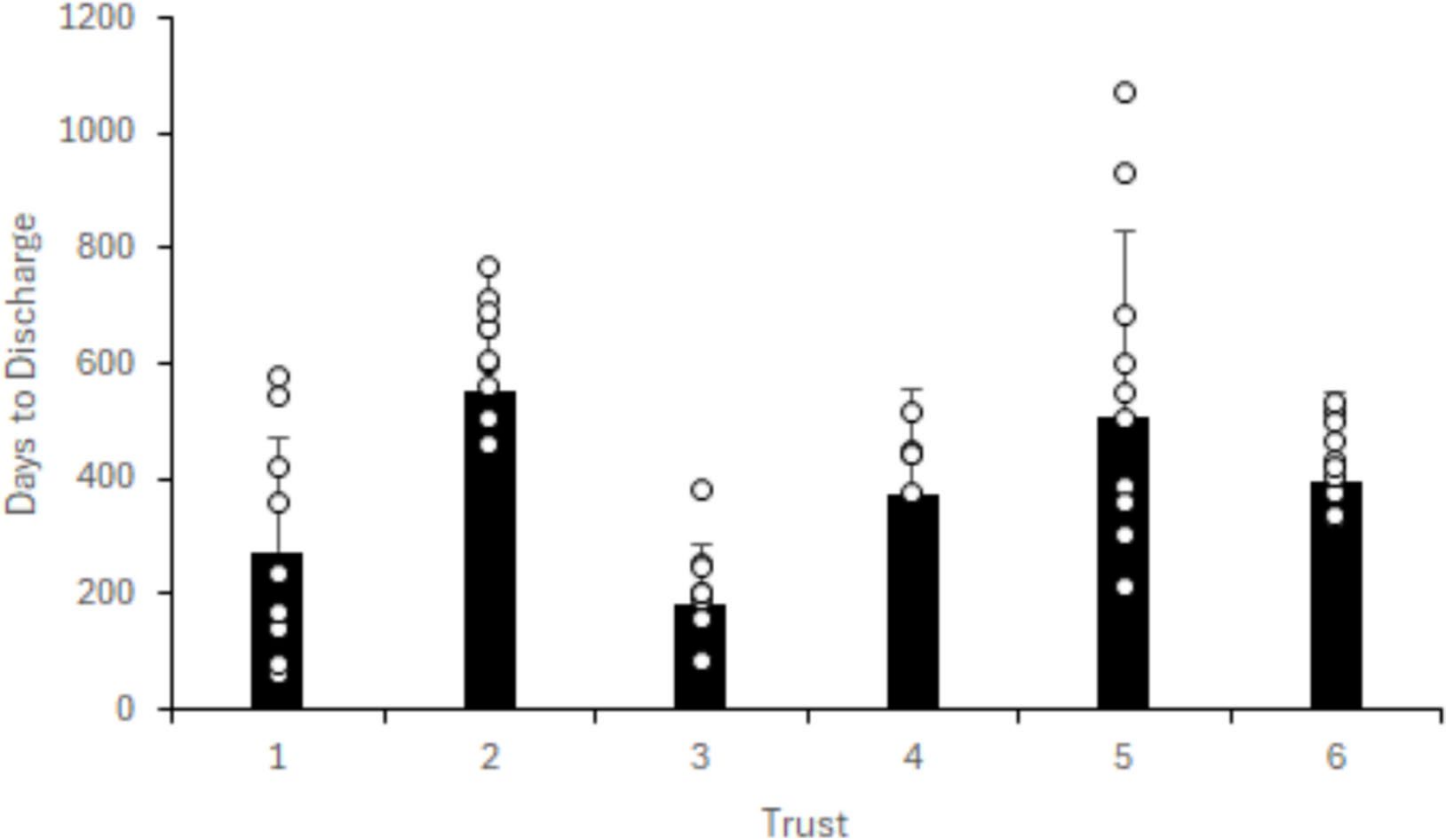
Number of days from first presenting to hospital until discharge. *° denotes each individual patient's numerical data point. For Trusts who presented data for ten patients, less data points are presented if one or more patients presented with the same numerical data point, Trust 4 present five patients*

Overall median time from injury to discharge from physiotherapy was 421 days (IQR 249 to 546) with Fig. 6 highlighting the individual number of days from first presenting to hospital to discharge per Trust. For the twelve patients who did not undergo operative management, eight (66.7%) did not complete their rehabilitation, leading to median time to discharge of 204 days (IQR 127 to 235). Of the forty-three who had surgery, 35/43 (81.3%) completed their rehabilitation leading to a median time to discharge of 495 days (IQR 383 to 588.5). With either operative or non-operative management, 16/55 (29%) patients failed to complete their rehabilitation. Taking RTS as a successful outcome, 27 (49.1%) patients who were injured during sport returned to their sport, whilst four (7%) did not.

## Discussion

This case series review outlines significant variation in care ACL injured patients received following MRI diagnosis across six NHS Trusts in the Yorkshire region. Specific variation between Trusts is highlighted in time to discharge, number of physiotherapy appointments conducted, number of days to surgical intervention, outcome measures used, and number of patients returning to sporting activity. However, good practice was indicated for early injury management, especially time to specialist review and MRI examination, both of which were consistent and acceptably quick across all Trusts.

### Patient and injury characteristics

Of the 55 cases reviewed, 75% were male (41:14), which is comparable with the 69% reported by the National Ligament Registry (2022). However, findings contrast with previous research which suggests that females are more likely to sustain a to ACL injury, with ratios ranging from 2:1 to 9:1 [36]. The exact difference in incidence among female athletes versus male athletes is not known, as incidence reporting in the research is often not sex specific. Our case review found a higher proportion of non-contact injuries (49/55), consistent with literature indicating non-contact mechanisms of injury as the most prominent in sports such as netball and soccer due to their pivoting nature [37]. This may partly be due to the availability and participation rates of female sports in some of the locales reviewed.

### Key pathway events

A positive finding is the relative ease that ACL injured patients navigated the treatment pathway and were able to access appropriate, prompt, and specialised care. The case review revealed an efficient initial treatment pathway with a median time to be seen by a specialist of 12 days; to receiving their MRI scan in 24 days from the injury being sustained. These results align with the objectives to see patients following acute knee injury within 14 days of the injury being sustained [38]. These results positively contrast with initial ACL injury management delays reported by a single NHS Trust with multiple sites [28], which may reflect the success of AKC implemented in the Yorkshire region [38]. Specialist AKC have been shown to positively influence the care pathway, enabling patients prompt access to specialised clinicians, timely access to MRI, and referral pathways to specialist consultants [38]. Getting it right first time (GIRFT) is a current national initiative, as suboptimal initial injury management has been shown to increase healthcare and economic costs [28]. As such our findings support previous research which has highlighted the benefits of adopting AKC in NHS settings [28, 39].

Variability in MRI wait times and subsequent follow-up (median 44 days) was observed, potentially due to multifactorial factors including clinic capacity pressures, in house prioritisation, protected MRI slots, and consultant availability. Surgical intervention times also highlighted variation (median 143 days; IQR 83.5–239 days), yet this is still quicker than the 300 days average previously reported in The National Ligament Registry (2022). The decision to operate considers associated injuries, patient demands, but can also allow time to ascertain whether a patient is a perceived ‘coper’ or ‘non coper’ with an ACL deficient knee [40]. Allowing time for rehabilitation and non-operative management may be beneficial as NHS wait times have increased post COVID- 19 pandemic, with 7.5 million people awaiting non-urgent elective treatment [41]. Delays in time to surgery could potentially be viewed as a positive, as more physiotherapy session can be undertaken which has been linked to better return to sport outcomes [42]. Furthermore, this supports suggestions that structured rehabilitation exceeding six months improves ACL injured patient’s opportunity of passing RTS criteria [20, 43]. As such, longer surgical wait times may have positive effects if accompanied by well-structured physiotherapy non-operative management.

### ACL injury management

Outcome measurements at regular intervals are advocated over time-based measures to guide ACL rehabilitation [44]. We found large variation in how different Trusts assess lower-limb strength for ACL injured patients. Methods of assessment included leg extension/press/curl gym equipment, handheld dynamometry, manual muscle testing, capacity testing (e.g., counting number of repetitions completed) and hop tests. Such variability in strength assessment use is consistent with previous research from other countries [26, 45]. Whilst no gold standard strength assessments exist for ACL injured patients, best practice guidelines and previous research suggest that limb symmetry index [23, 46, 47], and plyometric assessments/training [48] are important indicators of rehabilitation progression/regression. However, our review found only 55% of patients were assessed for LSI, comparatively, only 55% underwent plyometric training/testing. Hop tests for distance, though used in 50% of patient cases in the current review, has been identified as a poor measure of knee function in ACL injured patients and may obscure lower-limb limb biomechanics dysfunction [49]. Further to this, no Trusts, potentially due to geographical and/or economic factors, utilised isokinetic dynamometry despite widespread available contemporary research to validate its use when assessing strength [50, 51].

Outcome measurements were not undertaken and/or measurement recorded in approximately half of the patients reviewed. ACL injured patients should undergo stringent outcome assessments at regular intervals to determine the clinical efficacy and effectiveness of rehabilitation programmes [52]. Criteria driven rehabilitation is recommended to improve clinical outcomes, with patients required to meet key clinical criteria before progressing to the next stage [53]. Importantly, objective assessments can identify physical functional deficits, whilst providing opportunities for the clinician and patient to discuss how they will continue to work on these issues to avoid future pain and problems with the injured area [54]. Without the use of consistent outcomes, it becomes difficult to safely and effectively progress patients through rehabilitation to ensure they are ready for discharge. Our results suggest that outcome measures were being used to measure strength in some but not all patients, potentially hindering understanding of a patient’s rehabilitation progress and suitability for discharge.

Our findings question whether appropriate assessment methods are being utilised and if there is alignment between clinical practice and research. This issue is potentially further exacerbated due to clinician experience level, and/or time requirements spent on testing procedures, which may not be justifiable from an economic perspective [45]. Additionally, there is often a delay in implementing new evidence into clinical practice and a clinician’s willingness to adapt practice [45]. Henning et al. [53] suggested that manual input of patient data is time-consuming, potentially reducing the use of outcome measures in ACL injury management. Digital solutions could help by automating data capture and storage, potentially reducing the burden on clinicians, and increase the use of outcome measures. Research has shown that ACL injured patients highly value achieving relevant goals and milestones [54], whilst indicating that regular communication and supportive information creates a valuable opportunity to enhance the clinician/patient relationship, which has a positive correlation with patient outcomes [55].

Such variability in use or lack of use of outcome measures makes it difficult to compare results and understand when and how to progress patients. Svantesson et al. [52] used a modified Delphi method approach to planning optimal outcome assessment for ACL research studies highlighted four robust categories: early adverse events, PROMs, ACL graft failure/recurrent ligament disruption, and clinical measures of knee function and structure. With a little under half of the Trusts reviewed utilising PROMs further consistency is required if ACL injured patients are to be effectively and safely discharged.

### Rehabilitation and discharge

Although time-based methods are no longer advocated for assessing ACL injury recovery, research suggests delaying for a minimum of nine months post injury to allow an appropriate period of healing and return to function [3, 17]. This review suggests that most patients exceeded this timeframe with a median discharge from time of injury of 421 days (~ 14 months). However, duration is not the only criteria for successful discharge, with research highlighting frequency and adherence of rehabilitation to provide more favourable outcomes post ACL injury [56].

Physiotherapy was mostly delivered through a mixture of individual and group settings, with each Trust designing and implementing their own rehabilitation protocols. The number of physiotherapy sessions varied widely across Trusts, ranging from four sessions and seven out of ten patients not completing their rehabilitation, to another Trust with a median of 35 rehabilitation sessions, observing no dropouts. The latter Trust also used outcome measures with all their patients and successfully returned all to sport, suggesting that rehabilitation attendance is crucial to positive outcomes, improved knee function, reduced reinjury rates, and effective RTS [2, 3, 57].

Almost 30% of ACL injured patients included within the review failed to complete their rehabilitation, less than the 45% reported in previous research [58]. However, a novel finding of this study suggests that two thirds (8/12) of patients who did not undergo ACLR failed to complete their rehabilitation resulting in earlier discharge, compared with 19% (8/43) of those who received surgical reconstruction and exercise rehabilitation. Although based on a small sample, future research should investigate the associations between dropout rates and treatment pathways (operative and/or non-operative). Practitioners should engage patients with the decision-making process, seeking to understand their treatment preferences, values, and beliefs, whilst using contemporary evidence to educate patients and support an informed and shared decision-making process [28]. It was beyond the scope of the current study to investigate the reasons for discrepancies in rehabilitation adherence between surgically and conservatively managed patients. However, previous research highlights that greater patient adherence has been observed in ACLR patients when compared to non-surgical approaches [59]. It has been suggested that patients may perceive ACLR as a more serious intervention due to its invasive nature, leading to greater focus, time, effort, and willingness to engage in their rehabilitation [60]. Furthermore, ACLR patients may be more motivated to return to sporting activity, contributing to greater rehabilitation adherence rates due to the physical demands of sport and pre-established exercise habits [60]. Further research is required to determine whether similar discrepancies in adherence are observed in larger scale NHS based studies, where access to services and patient demographics may vary.

Long term follow up is lacking across Trusts, making it difficult to delineate the reasons for reattendance and give an indication of re-injury rates. Research indicates that ACL injured patients value discharge/return to sport tests for understanding physical deficits [54]. However, this review highlights that 27/55 patients did not receive specific discharge criteria, which can hinder clinician’s ability to make informed decisions, potentially leading to premature discharge, higher re-injury risks, lower rates of RTS [61] whilst also increasing the costs and complications associated with secondary ACL injury [62].

Our case review of ACL injury management across six NHS Trusts in the same geographical region reveals significant inconsistencies in the service and care provided to patients, supporting previous research findings at one NHS Trust across multiple sites [28]. Henning and colleagues [53] demonstrated how a single NHS provider improved the care they offer ACL injured patients by implementing a plan, do, study, act approach to address internal inconsistencies. This approach resulted in 100% of their patients participating in criteria-based progression rehabilitation, whilst RTS times were deemed to be in line with best-evidence recommendations [3, 17].

### Strengths

This is the first study to attempt to explore the ACL injury pathway across multiple NHS Trusts in the same geographical region, using a standardised operating procedure. Although a relatively small sample study, data suggests that there are discrepancies in patient care which need to be addressed if optimal patient outcomes are to be achieved. Findings of the current review, coupled with lessons from previous research including criterion driven rehabilitation implemented by trained core practitioners and using key stakeholders to drive change [53] could lead to improvements in ACL injured patient care throughout the Yorkshire region and beyond. Inconsistencies in care identified in the current study will help to inform a larger national scale study investigating ACL injury management pathways. With variations in service provisions, funding and staffing restrictions across NHS services, understanding optimal treatment is important to inform clinicians and financial stakeholders on future plans to improve ACL injury management, as services that lack clear guidance are likely to be cost inefficient [28].

### Limitations

This case series highlights variability in ACL injury, though it did not account for geographical and socioeconomic differences between NHS Trusts, including facilities, resources, staffing, and time allocated per patient. Furthermore, the authors acknowledge that whilst the data provides preliminary valuable insights regarding variable patient care in the region, it should be acknowledged the findings are based upon a limited sample size, are limited to NHS Trusts within the region analysed and do not provide any cross-Trust comparison. Future research needs to consider a larger, nationwide sample to determine a true reflection of ACL injury pathways in the NHS. While no patients in this study returned to services, long-term follow ups could provide insights into re-rupture rates and their impact on NHS costs and patient’s quality adjusted life years. Further research in this field could be used to guide improvements and help to develop easily implementable best practice guidelines for NHS settings.

## Conclusions

Our case series is the first study to explore the ACL injured patient’s journey from injury to discharge across multiple NHS Trusts in the same geographical region. This study builds upon single site studies and highlights good practice in the form of initial treatment pathways and use of AKC throughout the region, but also substantial variability in patient care from MRI diagnosis to patient discharge. Such inconsistencies can contribute to suboptimal outcomes for some patients. Whether treated operatively or non-operatively, for patients aiming to RTS, this was achieved with greater consistency when more physiotherapy appointments were undertaken, outcome measures and PROMs were used, and specific discharge criteria was utilised. Future larger pathway investigation studies incorporating causative and predictive analysis studies on a national scale are required to determine whether similar trends are observed in a wider ACL injured population, which could help to improve national pathways for patients and clinicians working towards ensuring more positive and standardised patient-related ACL injury outcomes.

## Data Availability

Data are available upon reasonable request to the corresponding author.

## Appendix 1

### Standard operating procedure sent to each site

#### Disparity in anterior cruciate ligament injury management: a case series review across six National Health Service trusts

Dear colleague,

As part of a research project looking at the local management of ACL injuries, we are carrying out an audit. This is in an attempt to describe the variation of what is deemed to be ‘standard’ care for patients with ACL injuries, managed either surgically or non-surgically and subsequently cared for in physiotherapy.

Currently there is no consensus amongst clinicians, both medical and non-medical, as to what the ‘gold-standard’ care is/should be. This audit/case series review will aim to describe the care given to patients at a number of centres in the Yorkshire region. We would be grateful for your support in undertaking a clinical audit to explore this in your Trust and have prepared the following criteria:

## Inclusion criteria

- Adults over the age of 16
- Physiotherapy led rehabilitation of ACL injuries (surgical and non surgical)

## Exclusion criteria

- –Multiligament injury with or without reconstruction involving both the ACL and PCL and reconstruction of either the LCL and/or the MCL.

## Sample required

- Please select a sample of 5 patients diagnosed with an ACL tear via MRI scan who have completed their course of treatment to discharge according to your locally held guidelines. This can be conservative or surgically led.
- To ensure randomisation of the sample collected, please use a consecutive sampling method. This means selecting consecutive patients from a selected time period. Their care should have commenced 3 months after your services were deemed to have returned to normal post the COVID- 19 closures. Please select the recognised date that your services resumed and move forwards on the calendar by 3 months, then select the next 5 patients that meet the inclusion criteria.
- Once 5 are selected as a pilot and sent over, we will ask for 5 more, totalling 10 from each site.

## Data management

Please obtain the relevant local approvals from your Trust for use in an audit and please ensure that all identifiable information is removed and anonymised prior to entering into the study. Any data received will be placed in a password protected file on a hospital Trusted server. Please ensure the least amount of people necessary access the data of the sample selected.

## Information required for case series review (please use the Excel spreadsheet attached)

**Table:**

Patient related:
Age at time of injury (years)
Sex
Injured during sporting activity
If sporting, please indicate type
Mechanism Contact v Non contact
Pathway related:
Injury date (if not known, please estimate dd/mm/yyyy)
Date of first assessment by ortho/APP/Physio
Time in days—Injury to contact with ortho/APP/Physio
Diagnosis made by
MRI date (dd/mm/yyyy)
Time in days—injury to MRI diagnosis
Date of first appointment post MRI to inform of results (dd/mm/yyyy)
Time in days—Injury to patient being aware of diagnosis
Other soft tissue injuries?
Operative v Non-Operative
Date of operation (dd/mm/yyyy)
Injury to Operation time in days
Management:
Was a protocol followed?
Did they attend classes?
Was plyometric testing used?
If yes, which tests, please list
Was limb symmetry index used and documented?
How was strength tested?
If other, please describe
Were any other adjuncts used? Eg BFS, TENS, FES, Acupuncture, GameReady
If yes, please list
Discharge:
Discharge date or date of last physio contact (dd/mm/yyyy)
Number of physio appointments
Time in days—Injury to discharge
Do you have specific discharge criteria? If yes, please detail in brief in the comments section below
Did the patient complete their rehab? (did they DNA)
Were patient reported outcome measures used?
If yes, please list
Did they return to sport?
Did they return to the service as they reinjured their ACL?
Any other comments, please add

### Any other comments

Please use this section for any information that you feel is pertinent or to declare any missing information. For example, if the patient has had a surgery but it is not listed in the drop-down menu.

*Drop Down options will be provided, please type further information where prompted.

### Cover sheet (please use attached document):

- Date(s) audit data obtained
- Name of person who completed the audit
- Sources used to collect date (medical notes, electronic notes, letters, etc.)

## Audit completion date: (Proposed _____)

### Standardisation

All data will be anonymised and it is our intention to publish this data, therefore, please ensure that this audit is registered and approved in your local NHS Trust.

Cases will be selected by a single identified person at each site and patient selection will be selected during an identified timeframe so as to avoid selection bias.

The opportunity for authorship will be offered to the person completing the audit; this will be subject to satisfying the criteria for authorship from the targeted journal.

If you would like to discuss the audit in any more detail or have any questions regarding completion of the audit, please do not hesitate to contact Niall Maher on contact information below.

Kind regards,

Mr. Niall Maher

Advanced Practice Physiotherapist

Chapel Allerton Hospital

Niall.maher@nhs.net

## Abbreviations

ACL: Anterior cruciate ligament
ACLR: Anterior cruciate ligament reconstruction
AKC: Acute knee clinic
LSI: Limb symmetry index
IKDC: International knee documentation committee
KOOS: Knee injury and osteoarthritis outcome score
MRI: Magnetic resonance imaging
NHS: National Health Service
PLYo: Physiotherapy lower limb Yorkshire working group
PROMS: Patient reported outcome measures
RTS: Return to sport
WYATT: West Yorkshire association of acute Trusts
